# Tinnitus referral pathways within the National Health Service in England: a survey of their perceived effectiveness among audiology staff

**DOI:** 10.1186/1472-6963-11-162

**Published:** 2011-07-06

**Authors:** Phillip E Gander, Derek J Hoare, Luke Collins, Sandra Smith, Deborah A Hall

**Affiliations:** 1 NIHR National Biomedical Research Unit in Hearing, 113 The Ropewalk, Nottingham, NG1 5DU, UK; 2 School of Clinical Sciences, The University of Nottingham, Nottingham, NG7 2RD, UK; 3 Division of Psychology, School of Social Sciences, Nottingham Trent University, Burton Street, Nottingham, NG1 4BU, UK

## Abstract

**Background:**

In the UK, audiology services deliver the majority of tinnitus patient care, but not all patients experience the same level of service. In 2009, the Department of Health released a Good Practice Guide to inform commissioners about key aspects of a quality tinnitus service in order to promote equity of tinnitus patient care in UK primary care, audiology, and in specialist multi-disciplinary centres. The purpose of the present research was to evaluate utilisation and opinions on pathways for the referral of tinnitus patients to and from English Audiology Departments.

**Methods:**

We surveyed all audiology staff engaged in providing tinnitus services across England. A 36-item questionnaire was mailed to 351 clinicians in all 163 National Health Service (NHS) Trusts identified as having a tinnitus service. 138 clinicians responded. The results presented here describe experiences and opinions of the current patient pathways to and from the audiology tinnitus service.

**Results:**

The most common referral pathway was from general practice to a hospital-based Ear, Nose & Throat department and from there to a hospital-based audiology department (64%). Respondents considered the NHS tinnitus referral process to be generally effective (67%), but expressed needs for improving GP referral and patients' access to services. 'Open access' to the audiology clinic was rarely an option for patients (9%), nor was the opportunity to access specialist counselling provided by clinical psychology (35%). To decrease the number of inappropriate referrals, 40% of respondents called for greater awareness by referrers about the audiology tinnitus service.

**Conclusions:**

Respondents in the present survey were generally satisfied with the tinnitus referral system. However, they highlighted some potential targets for service improvement including 1] faster and more appropriate referral from GPs, to be achieved through education on tinnitus referral criteria, 2] improved access to psychological services through audiologist training, and 3] ongoing support from tinnitus support groups, national charities, or open access to the tinnitus clinic for existing patients.

## Background

Tinnitus is the experience of sound without an external source and is often a chronic complaint. Studies on the prevalence of prolonged spontaneous tinnitus in adults have estimated its occurrence to be around 10% [1-3], with as many as 7% of adults in the UK seeking medical attention at some time during their adult life [4]. Scientific understanding of tinnitus is limited, but it is most often associated with hearing loss due to ageing or noise trauma [5,6]. Managing tinnitus is challenging because the condition is a symptom for which there is currently no available cure. Clinical management often requires input from multiple healthcare disciplines, which places a significant burden on the service [7-10]. From the perspective of the tinnitus patient, experiences with the healthcare system can appear uncoordinated leading to repeated consultations and delayed access to information, advice and intervention [11,12]. The National Study of Hearing reported that one third of people who had reported persistent tinnitus to their general practitioner (GP) were not referred to other NHS services [3]. A more contemporary survey indicated that the current situation has little changed [11]. While there are reports on the operation of individual tinnitus services within different countries [13-19], to our knowledge, there are no comprehensive surveys of the tinnitus referral system from the clinician's perspective.

An important factor contributing to effective management of tinnitus patients is a quick process of triage and referral to an appropriate professional. A rapid response is important because of the potential for serious health concerns, and the often high level of distress that tinnitus patients may present [20]. In 2006 the average waiting time for an initial audiology appointment in England was 18 weeks [21]. Sixty-six percent of patients waited more than 13 weeks and, in some Strategic Health Authorities, the average wait reached 45 weeks. The long waiting time within the NHS was addressed by the DH in 2007 when the target of a maximum 18 week wait was set for all patients, from the point of GP referral to treatment by the appropriate specialist [21]. This policy was introduced into the audiology specialty in a phased manner and 18-week targets were met by December 2008. In January 2009, the DH published a Good Practice Guide specifically for adults with tinnitus [22]. Its purpose was to inform commissioners and service managers about how to improve the service whilst still meeting the 18-week target. The Good Practice Guide recommends strategies for tinnitus assessment, management, and referral at four different levels of the service: primary care (GPs), local community-based tinnitus services (audiologists and hearing therapists), specialist hospital-based centres (multi-disciplinary teams that include audiologists, hearing therapists, ENT specialists, audiovestibular physicians, and clinical psychologists), and supra-specialist assessment centres (multi-disciplinary teams that can offer more complex audiological assessments, neurosurgical interventions, and radiotherapy). Patient routes through the system were to be determined by clinical assessment and specific referral criteria, designed with service efficiency and equity of patient care in mind. Details of Good Practice Guide referral criteria are discussed later.

Although we have heard anecdotal comments from clinician colleagues on the Good Practice Guide, the *national *impact of the DH recommendations is unknown. The goal of the present survey was to evaluate current practice with respect to the Good Practice Guide and gather opinions on the system of tinnitus care in England from the clinicians who see tinnitus patients in the Audiology Department. A companion paper reported responses to the subset of survey questions that referred to the clinical assessment and management of tinnitus *within *English Audiology Departments [23]. The main objective of the present paper is to report on those survey questions related to the processes of tinnitus patient referral, thereby evaluating the use of, and opinions on, current referral pathways *to *and *from *those Audiology Departments. We also discuss perspectives on the current NHS healthcare system as it relates to tinnitus and referral, namely challenges to its efficiency and the impact of the 18-week commissioning pathway.

## Methods

The following section provides a summary of the methods in the design, administration, and analysis of the audiology survey. A complete description of these methods is reported in the companion paper [23]. According to the NHS National Research Ethics Service ethical approval was not required for this service evaluation.

### Developing the questionnaire

Questionnaire development was directed by published guidelines for the design and conduct of survey research [24,25]. In brief, authors first generated a list of potential questions on 10 topics (18-week pathway, Good Practice Guide, referral process to and from audiology, specialist training, departmental staffing and resource management, assessment, treatment, outcome, support networks, and other). Questions were iteratively reviewed and modified, a process informed by two focus groups held with clinicians that specialised in tinnitus. The final 36-item questionnaire comprised 24 closed 'tick box' questions assessing practices and resources, and 12 open-response questions eliciting opinions of the service within the respondent's workplace and the system generally. See additional file 1: Survey questions for a list of the subset of questions specific to this report.

### Distribution

A database of NHS Trust audiology departments in England was compiled from mailing lists provided by the British Tinnitus Association and the Royal National Institute for Deaf People. Every audiology department was contacted by phone or email, and 351 individuals who manage or directly provide audiological services for people with tinnitus were identified by name. The questionnaire was then mailed to all 351 individuals, representing all 163 NHS Trusts across England identified with a tinnitus service.

### Data collection and analysis

Responses were entered into a database (Microsoft Access) and descriptive statistics and graphs were generated using Microsoft Excel. Statistical analyses were performed using the statistical software package R (2.11.1), with alpha level set to 0.05. Responses to some of the questions were further analysed statistically to test whether job role (i.e., audiologist, hearing therapist, or clinical manager) was associated with the response given.

### Thematic analysis of open responses

Open-question responses were subjected to a thematic content analysis [26]. This method codes and categorises sections of text based on their themes [26-28], and our specific protocol was based on Braun and Clarke [29]. Full details are provided in the companion paper [23].

## Results

We received 138 responses (39% response rate) from 42 hearing therapists, 80 audiologists, and one clinical psychologist. Fifteen respondents did not indicate their job title. Thirty-two clinicians described their role as including some form of management. In subgroup analyses these individuals were therefore categorised as 'clinical manager'. We identified 163 NHS Trusts across England which provided a tinnitus service, and received responses representing 73 separate Trusts distributed across all 10 Strategic Health Authorities (mean = 48%, range = 18-83% representation of Trusts across each Strategic Health Authority); 29 respondents did not identify their NHS Trust.

In the following sections, we describe responses to questions on the referral processes to the audiology department, from the audiology service, and the opinions on those processes elicited by the open questions. See additional file 1: Survey questions for a list of the subset of questions specific to this report. The responses to these questions address an independent set of survey items and do not overlap with the companion paper.

### Referral of the tinnitus patient to audiology

**Figure 1:**
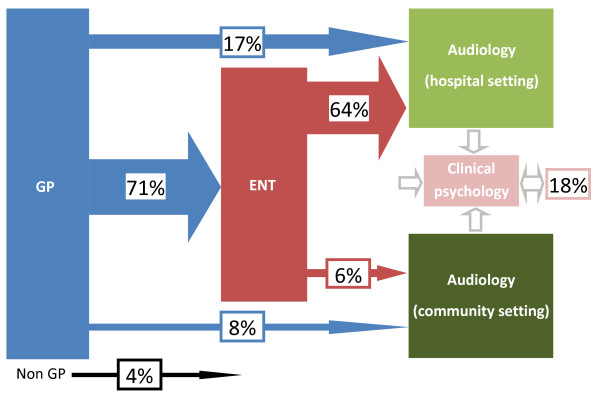
**Standard referral pathways for tinnitus patients**. The most commonly reported standard referral routes for tinnitus patients are shown. Note, not all pathways are shown. The directional arrows show the percentage of respondents who reported that their patients follow each stage of referral. Referrals to clinical psychology were reported to originate from any of the other clinical settings, and was included as part of the standard referral pathway in 18% of responses. The double-sided arrow indicates that in some cases the pathway continued on from clinical psychology to other medical services.

The most typical referral routes for tinnitus patients are shown in Figure 1. Almost all respondents (96%) indicated that the pathway began with the GP; for the remaining 4% the pathway began at the specialist hospital-based setting at audiology or ENT, where these patients are first identified as having tinnitus. The majority of pathways start with GP referral to an ENT department, and then from ENT onto a specialist audiology service in a hospital setting (64%). Six percent of respondents indicated that patients could be referred from ENT to a local audiology service in a community setting. Twenty-five percent of respondents reported that GP referral was direct to audiology (community-based = 8% and hospital-based = 17%). Hence, for a quarter of respondents, the 'typical' tinnitus referral pathway to their service bypassed ENT. Clinical psychology was identified to be part of the 'typical' pathway in 18% of responses, primarily from specialist audiology services.

In the UK, patients can access audiological services via their GP in one of two main ways, either by direct access (from GP to audiology), or by 'choose-and-book'. This latter route is an electronic referral service that gives patients the ability to select their first outpatient appointments in a community-based or hospital-based setting. When asked about how tinnitus patients access the ENT/audiology service from the GP (Question ii) just over one half of the respondents indicated both direct access and choose-and-book systems (56%), while the remaining responses were equally divided between direct access only (23%) or choose-and-book only (21%).

When asked how long it takes for a person with tinnitus to reach a tinnitus specialist in their local ENT/audiology service (Question iii), 45% of respondents indicated that it took between four and eight weeks. For 24% of respondents, it was less than four weeks, 20% indicated 8-12 weeks, and 8% 12-16 weeks. Three percent indicated that their waiting time for tinnitus specialist consultation was more than 16 weeks.

**Figure 2:**
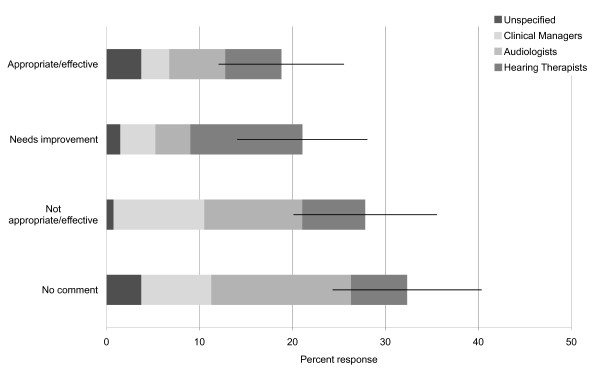
**Opinion on GP management of tinnitus**. Opinions on GP management of tinnitus patients are displayed according to the job role of respondents (the 95% confidence intervals are calculated for the responses pooled across all three job roles).

We asked clinicians how appropriately and effectively they believed local GPs manage people with tinnitus (Question iv). Based on the referrals they receive, clinicians expressed mixed opinions (Figure 2). Eighteen percent (25/138) considered GP management to be appropriate or effective, 20% (28/138) felt that it needed improvement, and 27% (37/138) felt that GP management was not appropriate or effective at present. Thirty-one percent (43/138) of respondents felt unable to comment on this question, possibly because they received referrals from ENT and did not have information about the original GP referral. Thematic analysis of the responses from those who felt that GP management was not appropriate or effective identified three themes: 1) GPs use an inappropriate intervention such as negative counselling (n = 14, e.g. "there is nothing that can be done for tinnitus"), 2) GPs delay or act as a barrier to referral (n = 11), and 3) GPs lack general knowledge of tinnitus and the management strategies available (n = 8). The job role seemed to influence the type of response given to Question iv (p = 0.024, two-tailed, generalised Fisher exact test (Fisher-Freeman-Halton)). Subsequent tests indicated that this effect was due to a greater proportion of audiologists than hearing therapists who commented that GP referral was not appropriate or effective, and a larger proportion of hearing therapists than audiologists that commented on the need for some improvement (p = 0.017, two-tailed Fisher exact test).

**Figure 3:**
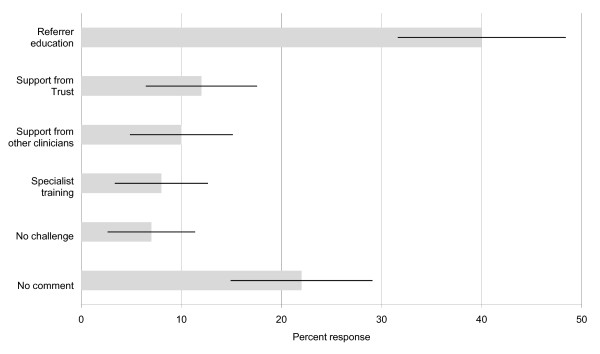
**Challenges to the efficiency of the referral process**. Results displayed are the percentage of respondents who identified each issue (± 95% confidence intervals). Referrer education included education of GPs and ENT specialists. A need to educate audiology staff on referral was not identified. Support from Trust included Trust managers and commissioners. Support from other clinicians included the other stages of the referral pathway and clinical psychology. Specialist training referred to specific training for audiologists on tinnitus management.

When asked about challenges to the efficiency of the referral process (Question viii, see Figure 3), 40% (53/131) of respondents indicated a general need to educate GPs and ENT specialists and raise awareness of tinnitus, the presence of tinnitus clinics, and the range of management strategies the clinics can offer. Of these 53 responses, 13 highlighted GP education specifically, 2 ENT education, and 5 indicated education of both GPs and ENTs. Inappropriate triaging of tinnitus patients due to a lack of education was also commented. Specific details included a lack of knowledge about the specific criteria for referral to audiology (n = 9), a lack of awareness about the services available at the tinnitus clinic (n = 8), and the general poor quality of tinnitus information given to patients (n = 4).

### Referral of the tinnitus patient from audiology

The Good Practice Guide outlines the services available at different levels of the tinnitus pathway. A 'specialist centre' is defined as one that provides medical, surgical, rehabilitative, psychological, and psychiatric services. However, it is not necessary for all services to be provided by the same department. In this sense a 'specialist centre' can be a virtual one. Only 10% of respondents indicated that all five services were available to them for onward referral, while 33% could refer to three or more services. There were differences in the number of respondents between Strategic Health Authorities who reported less than three compared to three or more services available (p = 0.05, two-tailed, generalised Fisher exact test (Fisher-Freeman-Halton)), with respondents from four Strategic Health Authorities indicating fewer options to refer to more than three services than those elsewhere. The services typically indicated as being unavailable were rehabilitative, psychological, and psychiatric.

Support from clinical psychology can be an important part of the treatment 'package' for some individuals who are severely debilitated by their tinnitus or who experience severe psychological problems such as depression and anxiety [30]. It has been recommended that referral to mental health professionals should be made when patients present with the potential for co-morbid conditions that indicate the need for further assessment [9]. The Good Practice Guide suggests that psychological screening be part of assessment at the specialist-level centre, in order to highlight whether psychological or psychiatric management is needed. Sixty-five percent of respondents indicated that they lacked the option to refer outside their ENT/audiology service to a clinical psychologist or other clinical specialist qualified in providing psychological therapy. It is interesting to note that fewer clinical managers (19%) indicated that clinical psychology services were available than did audiologists (38%) or hearing therapists (43%) (p = 0.024, two-tailed, Exact multinomial test). We return to this issue in the Discussion.

**Figure 4:**
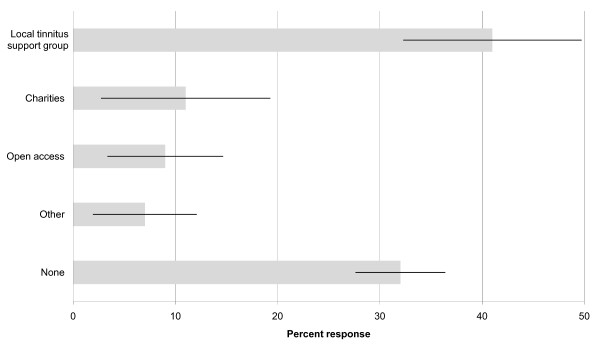
**Long-term support networks**. Responses are shown for the long-term support networks locally available for people with tinnitus. Responses are given as the percentage of respondents who indicated a type of support (± 95% confidence intervals).

It has been suggested that in only a few cases will people experience a spontaneous cessation of their tinnitus in response to treatment [31]. For many people, coping with tinnitus is a lifelong issue. We asked what long-term support networks were available locally for people with tinnitus (Question vii). Results from this open question are shown in Figure 4. While 41% of respondents indicated that there was a local tinnitus support group, 32% reported that that there was no such support available locally. Other sources of local support included non-tinnitus groups such as those for the hard-of-hearing (3%), for stress management (1%), and for lip-reading (1%). Telephone support and website information from relevant charities (including the British Tinnitus Association and the Royal National Institute for Deaf People) were mentioned by 11% of respondents. Open access to NHS ENT/audiology services after discharge was one possibility for continued support reported by 9% of respondents. The Good Practice Guide considers open access to mean either additional consultations or a telephone follow-up to assess the benefit of the hearing-aid fitting. The job role of respondents did not appear to influence the type of long-term support networks reported as available (p = 0.282, two-tailed, generalised Fisher exact test (Fisher-Freeman-Halton)), i.e. audiologists, hearing therapists and clinical managers were equally aware of their local services.

### Opinions about the referral process

Clinicians were asked to express their opinions about the referral process (Questions viii, ix) and identified a number of issues that they believe impact on its efficiency (see Figure 3). As already mentioned, one challenge is to educate those who refer to audiology services about the appropriate referral criteria (40%). A number of other challenges were identified. One was the lack of awareness from hospital administrators and service commissioners about the need to support a tinnitus service (12%). Another was the lack of support from healthcare professionals who operate within other stages of the pathway (10%). Interestingly, 8 of these 13 respondents highlighted an inability to refer or a difficulty in accessing clinical psychology. Another challenge identified was the lack of specialist training in tinnitus for audiology staff (8%). Seven percent of respondents did not feel that there were any particular challenges related to the referral process, while the remaining 22% did not provide comment. Job role was not found to influence the way in which these different factors were considered to impact on the tinnitus referral pathway (p = 0.466, two-tailed, generalised Fisher exact test (Fisher-Freeman-Halton)).

In general, respondents were positive about their local referral routes for people whose primary complaint is tinnitus. Forty-two percent (54/130) felt that the process was appropriate and effective. A further 25% (33/130) felt that the process worked but had some limitations. The main limitation was a breakdown in the referral pathway to the service (n = 19); either the GP (n = 15) or ENT consultant (n = 4) were felt to be dismissive of tinnitus complaints. The other major limitation was a breakdown in the pathway from the service (n = 14); either as a limited availability of psychological services (n = 11), or that referral back to the GP was required (n = 3). Eighteen percent (23/130) of respondents felt that the process was not appropriate or effective. Comments indicated that the referrals to the tinnitus clinic were sometimes delayed (n = 19), including referrals to ENT that were believed to be unnecessary according to referral criteria (n = 6), and others who commented that some referrals via a direct-access clinic could suitably bypass referral to ENT (n = 7). Five percent (6/130) of respondents felt that the referral process varied in how well it operated, and the remaining 11% (14/130) of respondents did not provide comment. Job role did not have an effect on the perceived effectiveness of the referral process (p = 0.768, two-tailed, generalised Fisher exact test (Fisher-Freeman-Halton)).

### 18-week commissioning pathway

**Figure 5:**
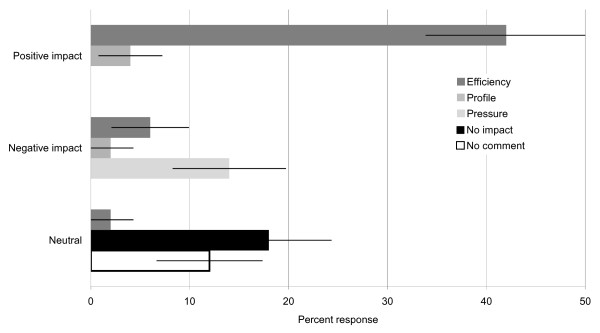
**Impact of 18-week pathway**. Opinions on the impact of the 18-week commissioning pathway are shown as proportion of responses in each thematic analysis category (± 95% confidence intervals). Themes are grouped according to whether the impact was positive, negative, or neutral. Also displayed are the responses for tick boxes indicating the inability to comment due to a lack of understanding of the pathway, or 'No impact'. A neutral impact on efficiency was noted by a small number of respondents.

We asked about the perceived impact of the 18-week commissioning pathway on the tinnitus service provided by audiology departments (Question x). The majority of respondents (70%, 98/141) felt that the pathway had impacted on their tinnitus service in some way, 18% (26/141) felt that there had not been any impact, while 12% (17/141) of respondents did not provide comment (Figure 5). Responses indicating there was an impact highlighted changes in service efficiency (n = 69), increased pressure (n = 21), and an increased awareness of their service as one in need of more resources (n = 8). Of those respondents who felt there was an impact 65 considered it to be positive, with improved efficiency resulting in a reduced referral-to-treatment time (n = 59). Some respondents indicated that it was critically important to be able to minimise wait time for those patients that were experiencing distress. Thirty-one respondents considered the impact of the 18-week pathway to be negative most commonly reporting increased time pressure (n = 20). For example, more patients were required to be seen within a smaller target time frame, leading to shorter appointment times. A minority of respondents (n = 8) indicated that the 18-week commissioning pathway raised the profile of the service (6 positive, 2 negative). Job role did not affect the perceived impact reported (p = 0.837, two-tailed, generalised Fisher exact test (Fisher-Freeman-Halton)), or whether the impact had a positive or negative effect (p = 0.754, two-tailed, generalised Fisher exact test (Fisher-Freeman-Halton)).

We also asked if the management of tinnitus appointments had changed since the 18-week pathway was introduced (Question xi). The majority of respondents (56%) stated that it had changed (we did not ask how), while 36% felt that it had had no impact. The remainder were not sure (8%). Again there were no significant differences in report according to job role (p = 0. 898, two-tailed, generalised Fisher exact test (Fisher-Freeman-Halton)).

## Discussion

The DH Good Practice Guide recommendations attempt to improve tinnitus services while maintaining a reduced referral-to-treatment time for NHS tinnitus patients of 18 weeks or below. The findings of this survey from approximately half of all services suggest that clinicians working in English NHS audiology departments generally consider the current tinnitus referral process to be effective. Nevertheless, our survey highlighted a number of key areas in which the current referral process and clinical practice in England did not quite meet the recommendations of the Good Practice Guide for adults with tinnitus. These are (1) more efficient referral from primary care, (2) better access to psychological services for those that need it, and (3) better continued support for patients after their initial management at the tinnitus clinic. We address each of these issues in more detail below.

### More efficient referral from general practice

The Good Practice Guide provides clear GP referral criteria. Patients with complaints of distressing tinnitus should be triaged either to local audiology services or to specialist centres, depending on the level of distress, complexity of the audiological profile and other medical, otological, or psychological factors co-morbid with their tinnitus. For a number of questions on the referral process, respondents to our survey were critical of GPs' management, expressing the opinion that GPs do not use the process of referral for tinnitus effectively and do not follow the appropriate referral criteria as outlined in the Good Practice Guide. There is an obvious need for education here. This may in part be addressed by audiologists themselves, in the form of explicit written feedback to referring GPs and ENT on the outcome of individual referrals.

GP management of tinnitus patients can have a large impact on the patient's outlook on tinnitus, often negatively as in the case of a patient who is distressed but is not referred on to other services [11]. A rapid response to onward refer distressed patients for appropriate psychological management at the specialist hospital-based centre is highlighted in the Good Practice Guide. However the typical pathway identified in our current survey is initial referral to an ENT consultant and thereafter referral to audiology and the tinnitus clinic where appropriate psychological management can begin. A few respondents commented that the visit to the ENT consultant before seeing an audiologist can be the wrong order of care in the pathway and increases referral-to-treatment times.

To assess the relative efficiency of tinnitus referral pathways, two pilot projects as part of the NHS Improvement programme were conducted in 2009-2010 on direct-access NHS tinnitus clinics in which the GP refers a tinnitus patient directly to audiology in a specialist centre. These two pilot sites were University Hospitals Birmingham NHS Foundation Trust - Selly Oak Hospital and Sherwood Forest Hospitals NHS Foundation Trust - Kings Mill Hospital. Reports on the pilot programmes indicate that referral-to-treatment times were considerably reduced, patient experience improved, appointment costs were lowered, and a large number of ENT outpatient appointments could be released [32,33]. From these reports, however, it is unclear if the baseline for comparison was the practice prior to the introduction of the 18-week commissioning targets. This would of course influence the degree of improvement reported. A critical factor determining the success of the direct-access approach was a greater GP awareness about the service through educational intervention [32,33]. In agreement with this conclusion, we also found that awareness and education of GPs about the tinnitus clinic and referral criteria were seen as one of the main barriers to an efficient tinnitus service. In the Good Practice Guide, initial assessment is recommended by a GP with a special interest in audiology/ENT, and if further triaging is necessary this could take place at a local audiology service by an appropriately trained clinician. The two direct-access pilots provide an example where local education and an increased awareness of the criteria for referring tinnitus patients directly to audiological services appeared to increase the availability of departmental resources and improve aspects of patient management.

Under the direct-access system audiologists need to be aware of symptoms that necessitate an otologic evaluation and referral to the ENT specialist for medical assessment, to rule out conditions such as an acoustic neuroma [9]. Like the Good Practice Guide in the UK, publications recommending clinical practice for tinnitus in the USA [9,34] state that audiologists who are the first contact for tinnitus patients should be aware of or typically have the training to identify the need for evaluation by a neuro-/otological specialist. Therefore, counselling and sound therapy approaches in the USA and Good Practice Guide in the UK do not necessitate an otologic evaluation by a neuro-/otologist for every tinnitus patient. However as mentioned by the authors of one guide for tinnitus practice it may be in the patient's best interest to receive such an evaluation [9]. A group of international researchers and clinicians forming the Tinnitus Research Initiative have recently created a tinnitus management guide which recommends that a specialised neuro-/otologist performs a clinical examination as a standard part of tinnitus diagnosis and therapeutic management [35]. It should be noted that the variations in recommendation across different guides for tinnitus management may be due to the specialism of the authors and differences in the healthcare systems where the authors of each guide are based [36]. The UK pilot programme for a direct access tinnitus clinic will need to address any potential issues with complications in the operation of the pathway, such as missed diagnoses, and report on outcomes from therapy, if it is to become a model for a new approach for tinnitus referral. Given the comments in the present survey about concerns for speed of referral and need for education on the pathway this may become a widely adopted approach to tinnitus management.

### Better access to psychological services for those who need it

The prevalence of co-morbid anxiety and depression has been found to be greater in chronic tinnitus patients compared to the general population [37-40], and the severity of tinnitus has been found to relate to the existence [8] and severity of depression or anxiety disorders [41]. As such some tinnitus patients may require the services of a psychologist, psychiatrist, or specialist counsellor [9,30,37,42]. Access to psychological and psychiatric services is identified in the Good Practice Guide as an important part of the tinnitus care pathway for the subset of patients requiring this service. However, responses to our survey suggest that access is limited and more access to psychology was desirable. The difference in reports on the availability of psychological services between clinical managers and audiologists and hearing therapists identified in this survey points to a need for education on the availability of such services. Inequities in service availability across the 10 Strategic Health Authorities were found for specialist level rehabilitative, psychological, and psychiatric services. The perception that not all services are available may reflect problems of variability in treatment availability or it may perhaps reflect some lack of knowledge of the onward services available. We do not have sufficient evidence to separate out these possibilities.

Difficulty in the access to psychological services has been identified in previous surveys on tinnitus service provision in the NHS [17,18], reflective of the lack of psychologists with a special interest in tinnitus and the limited resources of this service. Making psychological services a more mainstream part of the tinnitus pathway will be difficult without an increase in resources. To address the lack of access to clinical psychology, additional training in counselling and psychological therapies for clinicians in audiology has been recommended by the Good Practice Guide. However, this raises a question about what degree of training or counselling skills is appropriate. Moreover, training and provision of psychological therapies like cognitive behavioural therapy (CBT) for tinnitus within an audiology service tinnitus clinic also requires additional resources for training. CBT can also create a demand for greater clinic time, and will need professional supervision over therapy. While these resource implications will be difficult to meet for most departments, the availability of courses on psychological counselling may represent a compromise and lead to a better service for tinnitus patients. The training courses could prove effective by providing the audiologist with a skill set to better filter patients between either needing professional psychological help, or to services available at the audiology department, thereby improving the appropriateness of referrals to psychology, and reducing the overall amount of time a patient spends in the healthcare system. A study comparing CBT and a habituation-based treatment for tinnitus found that both interventions compared favourably in reducing tinnitus intrusiveness and distress [43]. However in this study the interventions were conducted by clinical psychologists. Presently, formal psychological treatment like CBT is best left to trained psychological professionals, as the effectiveness of CBT for tinnitus delivered by an audiologist or a hearing therapist has yet to be demonstrated [44].

### Better continued support after initial management at the tinnitus clinic

While the Good Practice Guide makes reference to self-management as a key initial stage of care by leaving responsibility with patients to obtain information on the condition and the variety of management options available [45], self-help is also an important part of long-term management [45-48].

The chronic nature of tinnitus means that some patients need continued support after discharge from audiology. A survey of tinnitus patients who received lay counselling at a tinnitus clinic found that 62% requested continued access to this service after the clinic follow-up appointment [17]. Only 9% of respondents in the present survey indicated that continued access to audiology was offered, despite the Good Practice Guide recommendation that long-term support at a local audiology service should be available to all. Again, this is possibly due to resource constraints or pressure from 18-week targets. Not all support needs to be provided by the NHS, in fact our survey showed that the most common form of long-term support was local tinnitus support groups, although many areas are not well served by this form of support. Charities and voluntary organisations are able to provide information and support for tinnitus, as mentioned in the Good Practice Guide, however, it is noteworthy that only 11% of respondents to our survey direct their tinnitus patients to charities.

## Conclusions

Our survey results are derived from clinicians working in English NHS audiology departments and apply directly to this health service. However, we encourage other researchers to conduct surveys of their tinnitus services to inform the international community about what are the strengths and weaknesses of different systems. By identifying themes regarding service provision we improve our ability to help the tinnitus patient.

According to those who responded to our survey the current tinnitus patient pathway is generally thought to be effective, and provides for the necessary management of most tinnitus patients in a timely manner. Improvements to the pathway can be achieved through GP education about referral criteria, through increased access to forms of specialised psychological support not available within the audiology department, and through the continued support of patients beyond the end of their initial management in the tinnitus clinic. In what is a time of flux in audiology and tinnitus services, the end goal being equity of service, this survey evaluation of current practice and opinion provides a baseline measure for changes in the referral process to come in the future.

## List of abbreviations

NHS: National Health Service; DH: UK Department of Health.

## Competing interests

The authors declare that they have no competing interests.

## Authors' contributions

PEG, DJH, and DAH contributed equally to this work by designing the study and analysing the data. PEG and DJH drafted the manuscript, while DAH contributed to the writing of the paper by revising it critically for important policy relevance and for intellectual content. LC and SS inputted and contributed to analysis of the data. All authors read and approved the final manuscript.

## Pre-publication history

The pre-publication history for this paper can be accessed here:

http://www.biomedcentral.com/1472-6963/11/162/prepub

## Supplementary Material

Additional file 1 **Survey questions**. Subset of survey questions used in this study. Click here for file
