# Supplementary material for: Tinnitus referral pathways within the National Health Service in England: a survey of their perceived effectiveness among audiology staff
Source: BMC Health Serv Res. 2011 Jul 6;11:162. doi: 10.1186/1472-6963-11-162 (PMC3144449; doi:10.1186/1472-6963-11-162)
Supplement: Additional file 1 — Survey questions. Subset of survey questions used in this study. [file 1472-6963-11-162-S1.PDF]

Additional file 1: Survey questions.

(i) In your local service, what is the standard referral pathway for people with tinnitus? Please number only the applicable options through the steps in the patient journey. Number them so that 1 = the first step, 2 = the next step, and so on.

____ GP

____ Audiology (hospital setting)

____ Audiology (community setting)

____ ENT specialty

____ Clinical psychology

____ Other medical specialty

(ii) How do tinnitus patients access your ENT/audiology service from their GP? Select all that apply.

Direct access via GP referral

Choose-and-book system

(iii) On average, how long does it take a person with tinnitus to reach the tinnitus specialist in your ENT/audiology service?

Less than 4 weeks

More than 4 but less than 8 weeks

More than 8 but less than 12 weeks

More than 12 but less than 16 weeks

More than 16 weeks

(iv) Based on the referrals that you receive, how appropriately/effectively do you believe your local GPs manage people with tinnitus?

____

I do not feel able to comment (additional tick box)

(v) Do you have the option to refer your tinnitus patients to other disciplines that offer a range of treatments? Select all that apply.

Medical

Surgical

Rehabilitative

Psychological

Psychiatric

(vi) Do you have the option to refer your tinnitus patients to a clinical psychologist or other specialist outside the ENT/audiology service who is qualified in providing psychological therapy?

Yes/No

(vii) What longer-term support networks are available locally?

____

(viii) What do you consider as the main challenge for implementing an efficient referral pathway for tinnitus care across different levels of your NHS network?

____

Do no feel able to comment (additional tick box)

No challenge (additional tick box)

(ix) From your point of view, how appropriate/effective is the referral process to and from your service for people whose primary complaint is tinnitus?

____

I do no feel able to comment (additional tick box)

(x) What do you consider has been the main impact of the 18-week commissioning pathway?

____

Do not understand the pathway in enough detail to comment (additional tick box)

No impact (additional tick box)

(xi) Has the management of patient appointments in your service changed as a result of the 18-week commissioning pathway?

Yes

No

Not sure

(xii) Your job title:
